# Effectiveness of an integrated cerebral protection protocol in type A aortic dissection surgery: an inverse probability treatment weighting analysis

**DOI:** 10.1186/s12893-025-02783-2

**Published:** 2025-02-11

**Authors:** Fei-min Shen, Yi-min Lin, Ming-cheng Huang, Jin-ping Liu, Ling-chen Huang, Liang-wan Chen, Xiao-fu Dai

**Affiliations:** 1 Department of Cardiac-thoracic Surgery, Zhangzhou Affiliated Hospital of Fujian Medical University, Zhangzhou, China; 2 Key Laboratory of Cardio-Thoracic Surgery, Fujian Medical University, Fujian Province University, Fuzhou, China; 3 Department of Surgery, Fuwai Hospital, National Center for Cardiovascular Diseases, Chinese Academy of Medical Sciences and Peking Union Medical College, Beijing, China; 4 Department of Cardiovascular Surgery, Union Hospital, Fujian Medical University, Fuzhou, China

**Keywords:** Cerebral protection, Type a aortic dissection, Inverse probability treatment weighting, Survival analysis

## Abstract

**Background:**

Cerebral protection strategies in type A aortic dissection (TAAD) surgery are critical yet inconclusive. We propose an integrated cerebral protection protocol. This study aimed to evaluate the effectiveness of this protocol.

**Methods:**

From January 2020 to December 2022, 85 patients were treated with an integrated protocol incorporating bilateral antegrade cerebral perfusion (ACP) and moderate hypothermia, with measures to prevent the shedding of thrombus or endothelial debris (BACP group), while traditional protocols were applied to 273 additional patients (UACP group). Inverse probability treatment weighting (IPTW) was performed to balance baseline characteristics. Three logistic regression models were used to evaluate the relationship between the two cerebral protection strategies and neurologic complications. Stepwise logistic regression was further employed to identify risk factors for cerebral complications.

**Results:**

Baseline characteristics were balanced after IPTW adjustment. The BACP group had a significantly shorter operative time (364.79 vs. 397.61 min, *P* = 0.022), significantly fewer neurologic complications (5.6% vs. 15.9%, *P* = 0.032), and transient neurologic injury (3.0% vs. 12.5%, *P* = 0.035). Binary multivariable logistic regression analysis showed that the cerebral complication risk was 3.14 times greater with the traditional protocol compared to the integrated protocol (odds ratio[OR]:3.14, 95%confidence interval[CI]:1.19–8.27, *P* = 0.020). Stepwise logistic regression confirmed that cerebral complications were dramatically increased with unilateral ACP (OR:2.99, 95%CI:1.14–7.82, *P* = 0.025), while bilateral ACP had a significant impact on decreasing cerebral complications.

**Conclusions:**

Our integrated protocol effectively minimizes postoperative cerebral complications. Moderate hypothermia combined with BACP and measures to prevent brain debris could be adopted as an effective strategy for cerebral protection in TAAD surgery.

**Supplementary Information:**

The online version contains supplementary material available at 10.1186/s12893-025-02783-2.

## Introduction

Type A aortic dissection (TAAD) is a life-threatening condition for which surgical intervention is the most effective treatment [1, 2]. For TAAD involving the aortic arch, total arch reconstruction with stented elephant trunk implantation is considered a well-established technique [3, 4]. However, temporary circulatory arrest is required to prevent backflow of blood from the lower-body to the operative field during the insertion of an elephant trunk stent and reconstruction of the aortic arch, leading to the necessity of addressing cerebral protection.

Cerebral protection during circulatory arrest is critical to patient prognosis but debate exists [5, 6]. Hypothermia and cerebral perfusion are widely accepted for cerebral protection [5]. Numerous studies have investigated the optimal core temperature and various cerebral perfusion strategies, including antegrade, retrograde, unilateral, and bilateral perfusion. Nevertheless, the discussion persists [7, 8].

Moreover, the high incidence of supra-aortic branches involvement, specifically the innominate artery [9, 10], coupled with frequent thrombosis within the false lumen and surgical manipulation that may result in dislodgment of thrombus or debris, dramatically increases the incidence of neurologic complications such as stroke, paralysis, and cognitive dysfunction.

In response to the aforementioned concerns, we implemented an integrated protocol for cerebral protection. This protocol involved using bilateral antegrade cerebral perfusion (ACP) with cerebral oximetry to evaluate cerebral oxygenation. Furthermore, moderate hypothermia was induced by maintaining a target temperature of approximately 26 °C. Finally, given the frequent involvement of supra-aortic branches with false lumen thrombus, precautions were implemented to prevent thrombus dislodgement into the cerebral artery and subsequent embolism. Additionally, an intra-aortic balloon occlusion (IABO) device was experimentally applied in the elephant stent to impede blood return from the descending aorta in some patients [11], thereby mitigating challenges associated with anastomosis and reducing circulatory arrest time. These improvements are expected to maintain effective cerebral perfusion during circulatory arrest, reduce time spent on cooling and rewarming, and prevent cerebral infarcts caused by medical factors, thereby reducing neurological complications.

We compared this integrated protocol with the traditional protocol used at our institution during the same time period. Inverse probability treatment weighting (IPTW) was performed to eliminate bias in baseline data. The main objective was to verify whether the integrated protocol could reduce the neurological complications after surgical repair and to assess its impact on clinical outcomes.

## Patients and methods

### Patients

**Fig. 1:**
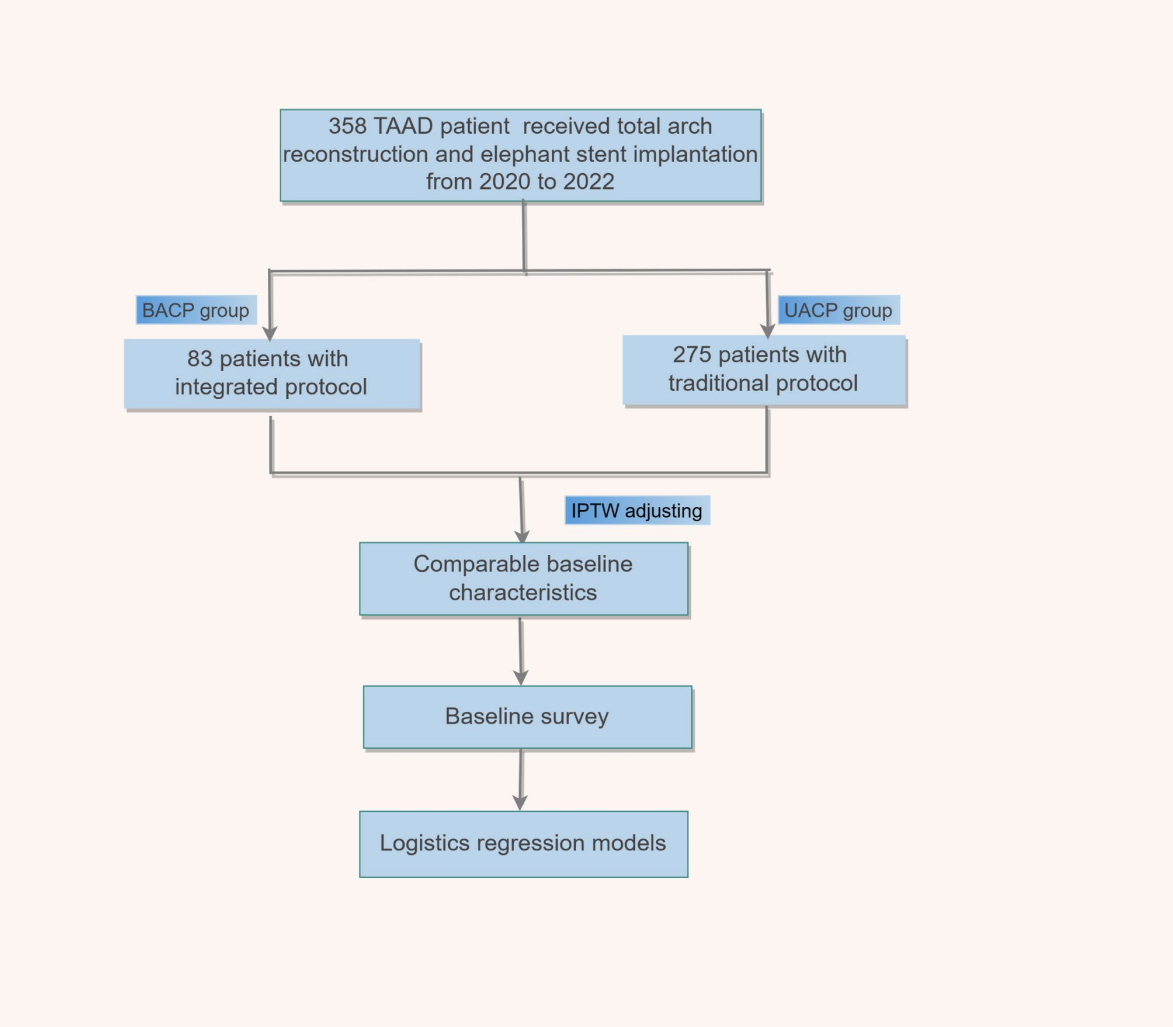
Study flow chart

From January 2020 to December 2022, a total of 358 consecutive patients who underwent total aortic arch reconstruction and stented elephant trunk implantation were enrolled at our institution. Ethical approval for this study protocol was obtained from the Institutional Review Board of the affiliated hospital of Fujian Medical University (approval ID: 2023KYB334), and written informed consent for the operation was obtained from all the participants and singed before surgery. 85 patients who received integrated cerebral protection protocol were included in the BACP group, while those 273 who received the traditional protocol were included in the UACP group. Participants should meet the following inclusion criteria: (1) conscious TAAD patients; (2) TAAD patients underwent stented elephant trunk implantation combined with total arch reconstruction. Exclusion criteria included: (1) patients with a history of stroke; (2) patients with symptoms of coma; (3) patients with cerebral malperfusion; and (4) patients who underwent only hemi-arch replacement or hybrid total aortic arch reconstruction. Figure 1 depicted the patient selection flow chart.

### Surgical technique

The surgical detail of TAAD repair has been described in detail previously [3, 4]. The difference lies in the cerebral protective measures employed during circulatory arrest.

In the BACP group, we performed BACP on all patients while using a moderate temperature of around 26 °C. Additionally, we used measures to sever the innominate artery in patients with suspected innominate artery involvement and combined false lumen thrombosis. We also attempted to reduce blood backflow from the descending aorta using the IABO technique in some patients in the BACP group, with the benefit of reducing circulatory arrest time. The IABO device was occasionally employed in the frozen elephant trunk in this group [2].

**Fig. 2:**
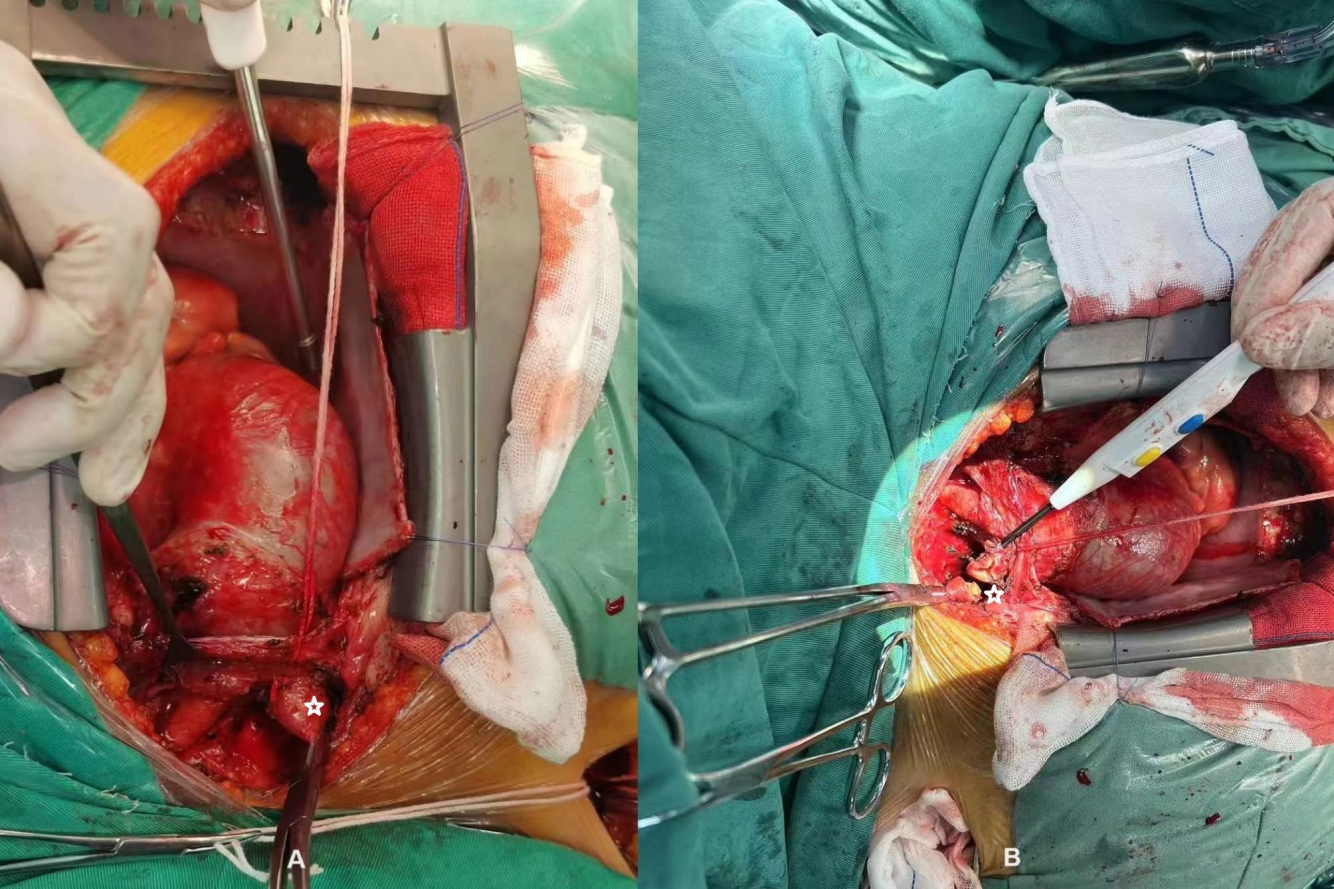
For TAAD involving the innominate artery or when there is a suspicion of a possible thrombus in the false lumen, the root of the innominate artery is clamped before initiating CPB (**A**), followed by severing the innominate artery (**B**). Pentagrams were used to label the innominate artery. TAAD: type A aortic dissection, CPB: cardiopulmonary bypass

The key points of the integrated cerebral protection protocol are as follows: ①Carefully preoperative assessment of the false lumen based on aortic CTA, evaluating the presence of thrombus, its location, size, and the risk of dislodgement; ②The right axillary artery was isolated and combined with the femoral artery for insertion of the arterial cannula. Intraoperatively, in patients with suspected thrombus in the false lumen of the aorta, meticulous manipulation was performed when severing the supra-aortic branches to avoid dislodgment of thrombus or debris in the false lumen. Before preparing to initiate cardiopulmonary bypass (CPB), the innominate artery was clamped at the root and severed to prevent thrombus from entering the brain (see Video 1 and Fig. 2); ③Before circulatory arrest, the left common carotid artery was severed, and an arterial perfusion cannula was inserted to conduct bilateral ACP; when circulatory arrest was achieved, the left subclavian artery was clamped to prevent stealing blood from the brain; ④Routine bilateral cerebral oximetry monitoring throughout the procedure, and the flow rate of cerebral perfusion was maintained at 10–12 ml/kg·min; ⑤Maintenance of core temperature around 26 °C (anogenital temperature) during circulatory arrest; ⑥Application of the technique of IABO experimentally in some patients: an Fr18 triple-lumen urinary catheter or a balloon catheter was inserted into the stented elephant trunk. The inflated balloon could prevent back blood flow in the descending aorta by occluding it, thus shortening the duration of circulatory arrest, which is important for high-risk patients [1].

Intraoperative and postoperative attention was paid to bilateral pupil diameters and pupillary light reflexes. High doses of sedative drugs were avoided during intensive care. The patient’s level of consciousness, neurological examination, and CT imaging findings were documented by reviewing the medical record system. Intraoperative and postoperative clinical parameters, such as operative time, ICU stay, hospital stay, and the occurrence of postoperative neurological complications, were also recorded.

### Definitions of clinical events and study endpoint

The Society of Thoracic Surgeons definitions were employed for post-operative complications. The primary adverse events included in-hospital mortality, cardiac shock, stroke, and hemodialysis [12].

To analyze neurological events after hypothermia circulatory arrest, we classified them into two categories: permanent neurological deficit (PND) and temporary neurological dysfunction (TND). PND was defined as postoperative presentation of symptoms of confusion, somnolence, and delirium without any neurologic signs and positive imaging findings. TND presents with symptoms such as coma and paraplegia, combined with positive neurologic signs and corresponding imaging findings that persist at the time of discharge.

### Statistical analysis

Statistical analysis was performed using R-studio with R 4.3.3, and statistically significant differences were defined as two-tailed P-values < 0.05. Due to differences in sample size and baseline characterize, IPTW was performed to minimize bias. The following baseline covariates were included in the matching process: age, gender, body mass index, hypertension, diabetes, coronary artery disease (CAD), smoke, alcohol consumption, bicuspid aortic valve, aortic regurgitation, left ventricular ejection fraction, preoperative serum creatinine levels, preoperative blood urea nitrogen levels, preoperative total bilirubin levels, preoperative alanine transaminase (ALT) levels, preoperative aspartate transferase (AST) levels, preoperative haemoglobin levels, preoperative platelet levels, and preoperative D-dimmer levels. Operative and postoperative data were compared between the two groups.

Three binary multivariable logistics regression models with forward stepwise addition of variables were applied to evaluate the association between cerebral protection protocols and neurological complications. Model 1 was univariate, model 2 incorporated age, gender, and cerebral protection protocol, and model 3 incorporated parameters such as age, gender, coronary artery disease, and cerebral protection protocol. Due to multicollinearity issues with surgical-related parameters, a stepwise regression model was applied to age, gender, CAD, and parameters of intraoperative cerebral protection measures (cerebral perfusion method, anal temperature, whether or not to sever the innominate artery) to screen out the risk factors.

## Results

### Baseline characteristics

**Fig. 3:**
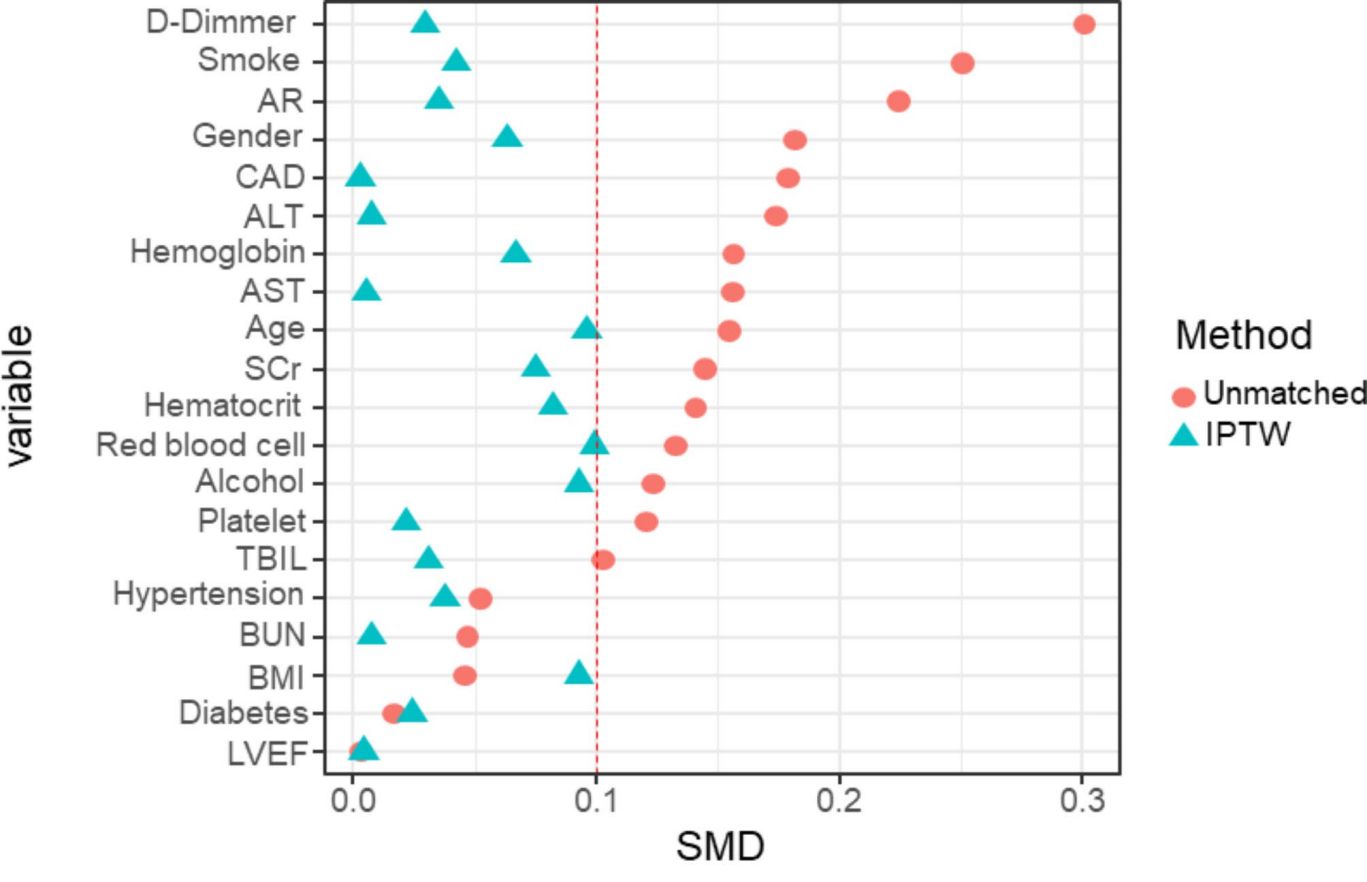
Standardized mean differences for baseline information between the two groups After weighting by IPTW, the SMD for all baseline variables were below 10%, showing that the variables were more comparable. IPTW: inverse probability of treatment weighting, SMD: standardized mean difference AR: aortic regurgitation, CAD: coronary artery disease, ALT: alanine transaminase, AST: aspartate transferase, SCr: serum creatine, BUN: Blood urea nitrogen, BMI: body mass index, LVEF: left ventricular ejection fraction

There were notable variances in certain baseline characteristics between the two cohorts. Specifically, the d-dimer level exhibited a statistically significant elevation in the BACP cohort compared to the UACP cohort (9.23 ± 8.46 vs. 11.67 ± 7.75 mg/L, *P* = 0.014). Additionally, there were indications of potential disparities in smoking history, preoperative ALT, and AST. To address these discrepancies, IPTW was utilized for baseline characteristic adjustment. As demonstrated in Fig. 3, the standardized mean difference (SMD) of all baseline variables was less than 10%, suggesting significant comparability between the two groups after IPTW adjustment. The demographic data of the study population were analyzed both before and after this adjustment, also shown in Table 1.

**Table 1:** Demographic and clinical data before and after IPTW matching

Item	Overall study population			IPTW Matched population		
	BACP	UACP	*P*	BACP	UACP	*P*
Number of patients	85	273		359	357	
Age (years), mean (SD)	52.22 (12.89)	50.31 (11.76)	0.202	49.48 (14.18)	50.73 (11.87)	0.600
Male, n (%)	62 (72.94)	220 (80.59)	0.176	273.0 (75.98)	280.6 (78.64)	0.637
BMI (kg/m²), mean (SD)	27.02 (4.51)	26.81 (4.63)	0.714	26.41 (4.51)	26.84 (4.61)	0.533
Hypertension, n (%)	68 (80.00)	224 (82.05)	0.790	287.3 (79.96)	290.7 (81.47)	0.784
Diabetes, n (%)	5 (5.88)	15 (5.49)	1.000	18.3 (5.09)	20.2 (5.66)	0.847
Coronary artery disease, n (%)	26 (30.59)	62 (22.71)	0.184	89.9 (25.02)	88.8 (24.89)	0.981
Smoke, n (%)	38 (44.71)	156 (57.14)	0.059	186.3 (51.85)	192.6 (53.98)	0.768
Alcohol, n (%)	31 (36.47)	116 (42.49)	0.390	131.5 (36.60)	146.8 (41.14)	0.512
Bicuspid aortic valve, n (%)	2 (2.35)	5 (1.83)	1.000	7.3 (2.03)	7.1 (1.99)	0.977
Aortic insufficiency, n (%)						
No regurgitation	32 (37.65)	107 (39.19)	0.351	142.0 (39.52)	137.7 (38.59)	0.995
Mild	24 (28.24)	55 (20.15)		81.5 (22.68)	78.6 (22.03)	
Moderate	14 (16.47)	63 (23.08)		73.4 (20.43)	77.4 (21.69)	
Severe	15 (17.65)	48 (17.58)		62.3 (17.34)	63.1 (17.68)	
Pre-LVEF (%), mean (SD)	60.65 (6.07)	60.67 (4.87)	0.976	60.66 (5.92)	60.64 (4.84)	0.974
Pre-SCr (µmol/L), mean (SD)	89.00 (47.75)	96.01 (48.93)	0.247	90.40 (36.25)	93.57 (47.35)	0.511
Pre-BUN (mmol/L), mean (SD)	7.48 (3.40)	7.67 (4.60)	0.724	7.62 (3.01)	7.59 (4.57)	0.947
Pre-TBIL (µmol/L), mean (SD)	17.60 (11.09)	16.58 (8.62)	0.376	16.43 (9.14)	16.71 (8.83)	0.791
Pre-ALT (U/L), mean (SD)	57.71(212.84)	30.81(50.63)	0.055	31.96(116.83)	31.25(52.71)	0.929
Pre-AST (U/L), mean (SD)	69.95(307.95)	34.52 (90.47)	0.093	34.75(163.65)	34.02 (85.80)	0.946
Pre-D-dimer (mg/L), mean (SD)	9.23 (8.46)	11.67 (7.75)	0.014	11.37 (8.70)	11.12 (7.75)	0.842
Pre-RBC (10^12/L), mean (SD)	4.24 (0.53)	4.45 (2.13)	0.377	4.27 (0.52)	4.41 (2.01)	0.266
Pre-Platelet (10^9/L), mean (SD)	197.24 (90.56)	187.26 (73.83)	0.304	188.44 (82.09)	190.17 (75.56)	0.867
Pre-Hematocrit (%), mean (SD)	38.46 (4.70)	39.13 (4.73)	0.258	38.54 (4.61)	38.92 (4.74)	0.535
Pre-Hemoglobin(g/L), mean (SD)	129.33 (17.46)	132.00 (16.65)	0.203	130.10 (17.38)	131.24 (16.75)	0.625

IPTW: Inverse probability of treatment weighting, BMI: Body Mass Index, LVEF: Left Ventricular Ejection Fraction, SCr: serum creatinine, BUN: blood urea nitrogen, TBIL: total bilirubin, ALT: alanine Transaminase, AST: aspartate Transaminase

### Analysis of surgical and postoperative notes between the two groups

As indicated in Table 2, after IPTW adjustment, notable variations exist in the parameters of the cerebral protection protocol, encompassing antegrade cerebral perfusion measures, core temperature management, and the decision to sever the supra-arch branches (*P* < 0.001). However, since not all patients have combined false lumen thrombosis or innominate artery involvement, the innominate artery did not routinely sever in the BACP group. Additionally, it is clearly that the total operative time is significantly reduced in the new strategy group (364.79 vs. 397.61 min, *P* = 0.022), and CPB time (181.49 vs. 193.18 min) also showed a decreasing trend.

**Table 2:** Intra-operative data before and after IPTW matching

Item	Overall study population			IPTW Matched population		
	BACP	UACP	*P*	BACP	UACP	*P*
Number of patients	85	273		359	357	
Cerebral perfusion strategy, n (%)			< 0.001			< 0.001
BACP	85 (100.00)	0 (0.00)		359.3 (100.00)	0.0 (0.00)	
UACP	0 (0.00)	273 (100.00)		0 (0.00)	356.8 (100.00)	
Core temperature (℃), mean (SD)	26.29 (1.73)	25.04 (2.31)	< 0.001	26.20 (1.84)	25.09 (2.36)	< 0.001
Innominate artery severed, n (%)	77 (90.59)	0 (0.00)	< 0.001	327.6 (91.18)	0.0 (0.00)	< 0.001
Operative time (min), mean (SD)	368.68(102.34)	398.59 (113.31)	0.030	364.79(100.43)	397.61 (112.18)	0.022
CPB time (min), mean (SD)	185.01 (76.85)	193.59 (70.68)	0.340	181.49 (77.55)	193.18 (69.74)	0.257
Cross-clamp time (min), mean (SD)	121.53 (53.71)	120.10 (44.67)	0.806	124.00 (57.78)	119.90 (43.91)	0.609
HCA time (min), mean (SD)	17.49 (23.00)	14.93 (5.60)	0.091	19.70 (28.71)	14.88 (5.64)	0.226

IPTW: Inverse probability of treatment weighting, BACP: Bilateral anterograde cerebral perfusion, UACP: Unilateral anterograde cerebral perfusion, CPB: cardiopulmonary bypass, HCA: hypothermic circulatory arrest (lower extremity only)

As shown in Table 3, there was a significant reduction in the incidence of TND (3.0% vs. 12.5%, *P* = 0.035) and a significant reduction in overall neurologic complications (5.6% vs. 15.9%, *P* = 0.032), demonstrating the effectiveness of our protocol. Although the circulatory arrest time demonstrated a trend toward an increase, no statistically significant differences were seen in the indices reflecting visceral ischemia, such as the postoperative CRRT rate and the incidence of paraplegia.

**Table 3:** Postoperative data before and after IPTW matching

Item	BACP	UACP	*P*	BACP	UACP	*P*
Number of patients	85	273		359.3	356.8	
MACE, n (%)	5 (5.88)	22 (8.06)	0.668	27.3 (7.60)	27.9 (7.82)	0.957
IABP, n (%)	1 (1.18)	5 (1.83)	1.000	2.3 (0.64)	6.1 (1.71)	0.360
ECMO, n (%)	1 (1.18)	4 (1.47)	1.000	5.8 (1.61)	5.0 (1.40)	0.908
Cerebral complications, n (%)	5 (5.88)	43 (15.75)	0.032	20.1 (5.59)	56.9 (15.95)	0.032
PND, n (%)	2 (2.35)	11 (4.03)	0.697	9.2 (2.56)	14.6 (4.09)	0.536
TND, n (%)	3 (3.53)	34 (12.45)	0.031	10.9 (3.03)	44.6 (12.50)	0.035
CRRT, n (%)	9 (10.59)	29 (10.62)	1.000	41.2 (11.47)	36.9 (10.34)	0.792
Pneumonia, n (%)	16 (18.82)	56 (20.51)	0.854	73.0 (20.32)	72.5 (20.32)	1.000
Ventilation time (hours), mean (SD)	46.40(47.41)	50.94(122.17)	0.738	47.89 (52.37)	50.20(122.71)	0.821
ICU stay (days), mean (SD)	6.31 (5.16)	6.21 (5.37)	0.883	6.35 (5.31)	6.17 (5.48)	0.818
Hospital stays (days), mean (SD)	14.14 (6.33)	14.51 (8.05)	0.697	14.04 (6.24)	14.62 (8.25)	0.537

IPTW: Inverse probability of treatment weighting, MACE: major adverse cardiac events, IABP: intra-aortic balloon pump, ECMO: extracorporeal membrane oxygenation, PND: permanent neurological deficit, TND: temporary neurological dysfunction, CRRT: continuous renal replacement therapy, ICU: intensive care unit

**Fig. 4:**
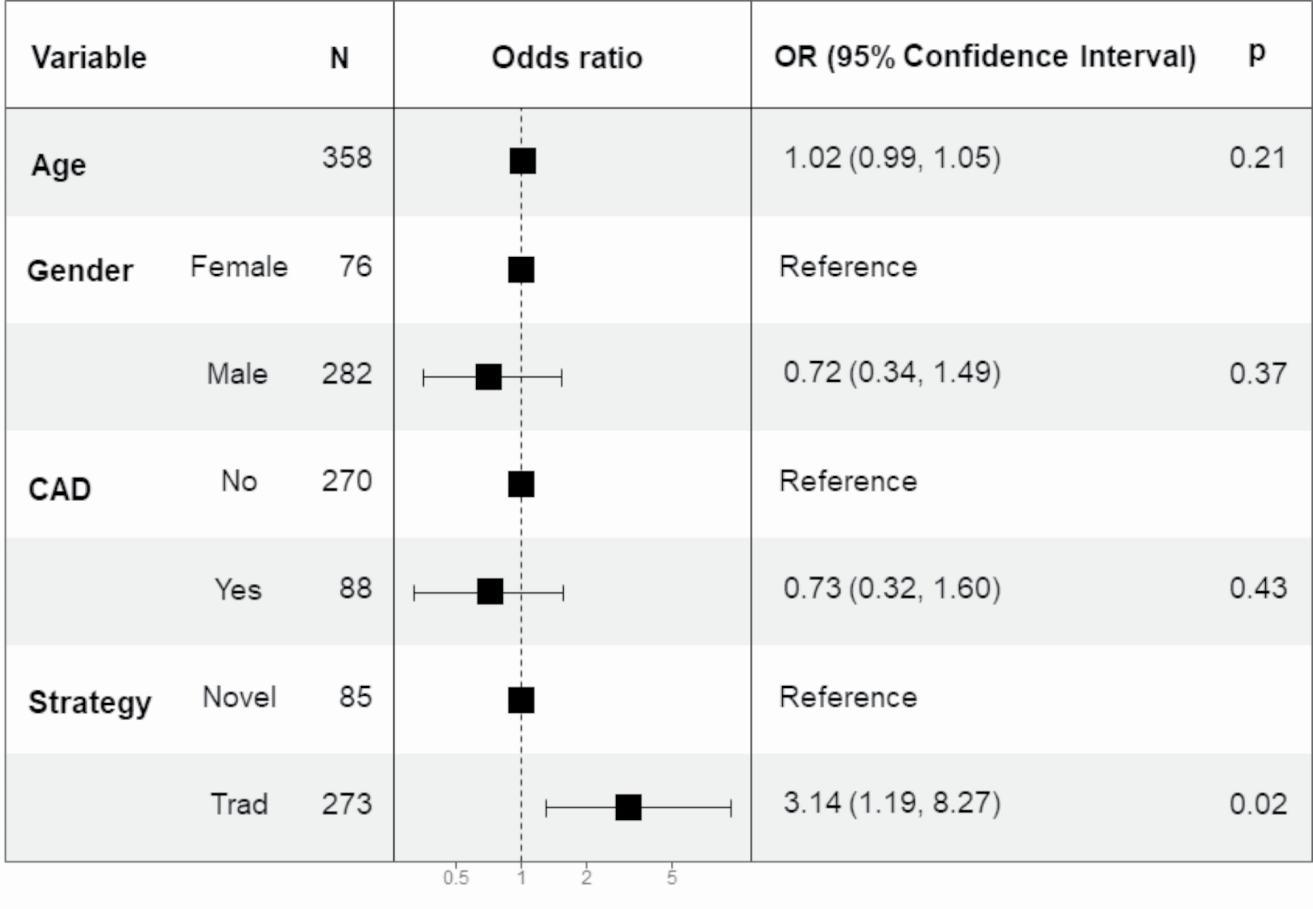
Forest map of multivariable logistics regression analysis of overall neurological complications. It demonstrated that the integrated cerebral protection protocol was strongly negative associated with overall neurological complications CAD: coronary artery disease, OR: odds ratio

The findings from the three regression models were as follows: model 1 (odds ratio [OR]: 2.99, 95% confidence interval [CI]: 1.15–7.82, *P* = 0.025), model 2 (OR: 3.20, 95%CI: 1.22–8.42, *P* = 0.018), and model 3 (OR: 3.14, 95%CI: 1.19–8.27, *P* = 0.020), indicating a significant negative association between our integrated cerebral protection protocol and neurological complications across all models. These results affirm the validity of the study’s conclusions. Specifically, in model 3, it was observed that the traditional protocol was associated with a 3.14 times higher risk of postoperative cerebral complications compared to our novel protocol, as illustrated in Fig. 4.

The relationship between each specific measure of cerebral protection and postoperative neurological complications was also evaluated. Since the method of cerebral perfusion, core body temperature, and whether or not to sever the innominate artery were highly correlated with the integrated protocol, there was a problem of multiple covariance. Thus, we used stepwise logistic regression, and the results showed that UACP has a 2.99-fold risk of cerebral complication compared to BACP (OR:2.99, 95%CI:1.14–7.82, *P* = 0.025), as shown in Table 4.

**Table 4:** Results of stepwise multivariate logistics regression analysis (with cerebral complications as the dependent)

Cerebral complications		No (*N* = 310)	Yes (*N* = 48)	OR and 95% CI (Univariable regression)	OR and 95%CI (Stepwise regression)
Age, per 1 year	Mean ± SD	50.5 ± 11.9	52.6 ± 12.7	1.01 (0.99–1.04, *P* = 0.262)	
Sex	Female	63 (20.3%)	13 (27.1%)		
	Male	247 (79.7%)	35 (72.9%)	0.69 (0.34–1.37, *P* = 0.288)	
Coronary artery disease	No	232 (74.8%)	38 (79.2%)		
	Yes	78 (25.2%)	10 (20.8%)	0.78 (0.37–1.64, *P* = 0.518)	
Core temperature, per 1℃	Mean ± SD	25.3 ± 2.3	25.6 ± 2.2	1.05 (0.92–1.20, *P* = 0.456)	
Innominate artery severed	Yes	72 (23.2%)	5 (10.4%)		
	No	238 (76.8%)	43 (89.6%)	2.60 (0.99–6.81, *P* = 0.052)	
Cerebral perfusion strategy	BACP	80 (25.8%)	5 (10.4%)		
	UACP	230 (74.2%)	43 (89.6%)	2.99 (1.14–7.82, *P* = 0.025)	2.99 (1.14–7.82, *P* = 0.025)

OR: Odds ratio, CI: Confidence interval, BACP: Bilateral anterograde cerebral perfusion, UACP: Unilateral anterograde cerebral perfusion

## Discussion

Our study demonstrated that this integrated protocol is a feasible and simple method to reduce neurological complications in TAAD patients who underwent stented elephant trunk implantation and total arch reconstruction.

Surgical treatment has dramatically reduced the mortality rate of TAAD [1]. The total arch reconstruction is currently the predominant procedure for TAAD involving the aortic arch. However, TAAD is still associated with a higher incidence of cerebral complications. During deployment of the stented elephant trunk into the true lumen of the descending aorta and subsequent anastomosis of the distal portion of the graft as well as anastomosis of the supra-aortic branches, circulatory arrest is required and therefore inevitably involves the issues of cerebral protection. The etiological mechanisms of neurologic complications in TAAD patients are multifactorial and is usually associated with circulatory arrest, systemic hypoxia and thromboembolism originating from the false lumen. We concluded the risk of neurological complication into three sources: (1) measures taken for cerebral perfusion and cerebral protection; (2) duration of cerebral ischemia and hypoxia; and (3) iatrogenic cerebral embolism.

Our integrated cerebral protection protocol proved to be an effective and safe way to reduce the incidence of postoperative cerebral complications without increasing in-hospital mortality. According to the logistics regression models, our integrated protocol was negatively correlated with cerebral complications. Furthermore, the stepwise regression analysis revealed that BACP is a strong protective factor. Additionally, the shortening of the operative time, which we believe may be related to the higher core body temperature. Maintaining the target core temperature at 26 °C significantly reduced the cooling and rewarming time compared with the conventional approach with deep hypothermia at 21 °C. Moreover, the CPB time also showed a trend of shortening, further confirming that the temperature we set shortened the time needed for cooling and rewarming, consequently resulting in the shortening of the operative time.

There is no conclusive consensus on different strategies of cerebral perfusion. Retrograde cerebral perfusion is currently not the mainstay of cerebral protection due to its obvious drawbacks (13–14). Previous literature has confirmed that BACP allows for longer perfusion duration compared to unilateral ACP [15]. As verified in our results, BACP proved to be a reliable approach for cerebral protection.

There is also no definitive conclusion on the optimal temperature for cerebral and spinal protection. Some scholars arguing that deep hypothermia remains the gold standard [16]. However, deep hypothermia poses problems such as coagulation dysfunction, prolonged operative time and CPB time, and the need for more blood transfusions, which are recognized risk factors for postoperative renal failure. Additionally, with the introduction of selective cerebral perfusion techniques for aortic arch surgery, it has been recommended to maintain temperature at a moderately hypothermic level [17]. Tsai compared moderate to deep hypothermia with total arch replacement using ACP and found that moderate hypothermia was associated with lower 30-day mortality and morbidity compared to patients who received deep hypothermia circulatory arrest [18]. As a high-volume centre with sophisticated surgical techniques and routinely use ACP, it would be more advantageous to set the temperature at moderate hypothermia, which is approximately 26 °C in our institution.

Researches also have noted that thrombus from a thrombosed false lumen is an important cause of postoperative neurologic complications [19]. Thrombosis of the supra-aortic branches, especially when the false lumen of the innominate artery is involved, is associated with a high risk of neurological complications [20]. The involved innominate artery and a thrombosed false lumen, often leading to thromboembolism from shedding thrombi, have been identified as significant contributors to postoperative neurological complications [19, 21]. Therefore, carefully and meticulously manipulation is essential in minimizing the risk of iatrogenic embolization.

The risk of brain tissue ischemia, anoxic necrosis during circulatory arrest is no longer a huge issue due to the utilize of ACP and even BACP. Our study confirmed that moderate hypothermia combined with selective cerebral perfusion reduces the cooling and rewarming time as well as the total operative time. However, this approach inevitably poses the problem that higher core temperatures lead to a reduced tolerance to lower body ischemia. With the new strategy involving manipulations such as IABO,, there is a tendency for prolonged circulatory arrest time. However, the incidence of CRRT and paraplegia, as indicators of spinal cord ischemia and abdominal visceral ischemia did not differ significantly between the two groups in our study, which reinforces that the only potential drawback of this strategy, namely the prolongation of lower-body ischemia, did not lead to a significant increase in the associated risk. This confirms the safety of our integrated cerebral protection protocol.

Although the clinical results demonstrate the superior efficacy of this cerebral protection protocol, there are several limitations to our study. The sample size was limited, and further follow-up is required to gather a larger cohort for assessing long-term outcomes. Despite adjusting for many potential confounders, residual confounding remains a possibility due to the observational design. However, despite these limitations, this protocol has proved to be effective for reducing in-hospital neurological complications, and in view of its simplicity and ease of operation, it can be recommended for further applied.

## Conclusions

This integrated protocol is safe and effective for cerebral protection, with good in-hospital results in preventing and reducing neurological complications during TAAD surgery.

## Electronic supplementary material

Below is the link to the electronic supplementary material.

Supplementary Material 1

## Data Availability

Anonymous raw data are available from the first author and corresponding authors upon agreement of the institutional ethics committee and with reasonable request.

## Abbreviations

TAAD: Type A aortic dissection
ACP: Antegrade cerebral perfusion
IABO: Intra-aortic balloon occlusion
IPTW: Inverse probability of treatment weighting
PND: Permanent neurological deficit
TND: temporary neurological dysfunction
CAD: Coronary artery disease
ALT: Alanine transaminase
AST: Aspartate transferase
SMD: Standardized mean difference
